# Usefulness of the maximum standardized uptake value for the diagnosis and staging of patients with cervical cancer undergoing positron emission tomography/computed tomography

**DOI:** 10.1097/MD.0000000000009856

**Published:** 2018-02-16

**Authors:** Hiroaki Takagi, Jinichi Sakamoto, Yasuhiro Osaka, Takeo Shibata, Satoko Fujita, Toshiyuki Sasagawa

**Affiliations:** Department of Obstetrics and Gynecology, Kanazawa Medical University, School of Medicine, Ishikawa, Japan.

**Keywords:** cervical cancer, Positron emission tomography/computed tomography, standardized uptake value

## Abstract

Cervical cancer recently has become more common among younger women in Japan. Diagnosing early-stage cancer is straightforward using cervical cytodiagnosis and histological diagnosis. However, postmenopausal endophytic cervical cancer and skip lesions in cervical adenocarcinoma are difficult to detect. We compared the maximum standardized uptake value (SUVmax) of 18F-fluorodeoxy-glucose positron emission tomography/computed tomography (PET/CT) for primary staging of cervical cancer and evaluated the relationship of the imaging findings to prognosis.

This was a retrospective study of 38 patients with cervical cancer who underwent PET/CT. Patients were grouped according to disease stage, and the mean SUVmax, overall survival, and progression-free survival (PFS) were evaluated.

The mean SUVmax was significantly different between patients with stage ≤I and ≥II diseases among those with squamous (*P* > .001) and glandular (*P* = .023) lesions. With an SUVmax of receiver operating characteristic curves as the optimal cutoff value, the log-rank test for PFS revealed a statistically significant difference between the 2 disease stages (*P* = .020 and *P* = .016, respectively).

SUVmax is useful to differentiate between stage ≤I and ≥II cervical cancer. SUVmax may be useful for the prognostic evaluation of disease recurrence in patients with cervical cancer.

## 1 Introduction

Cervical cancer is diagnosed clinically by analysis of tumor markers, ultrasound examination, computed tomography (CT), and magnetic resonance imaging (MRI). However, diagnostic precision remains inadequate. Recently, positron emission tomography (PET) has been used widely as a high-precision method of diagnosing gynecologic cancer. In 1956, PET studies by Warburg demonstrated that cancer cells use glucose in large quantities.^[1]^ Glucose uptake is 3 to 8 times greater in cancer cells than in normal cells.^[2,3]^ Glucose uptake in cancer cells is determined using 18F-fluorodeoxy-glucose (FDG), which is a radioactive tracer used during PET imaging, and is measured using the maximum standardized uptake value (SUVmax), which is a digital representation of significant FDG accumulation in tissues. Furthermore, PET/CT has a higher accuracy than separate PET and CT scans read side by side.^[4]^ PET/CT characteristics are suitable for the early detection of cancer, differential diagnosis of benign and malignant diseases, diagnosis of areas and spread of cancer and evaluation of treatment effect.^[5]^ PET/CT for the diagnosis of cervical cancer stage ≥ IB has a high sensitivity and specificity.^[6]^ It also has a favorable diagnostic value for distant metastasis. In addition, PET/CT scans are valuable tools in suspected recurrent cervical cancer cases.^[7]^

Recently, cervical cancer has become common among younger women in Japan.^[8]^ Diagnosing early-stage cancer is straightforward using cervical cytodiagnosis and histological diagnosis. However, postmenopausal endophytic cervical cancer and skip lesions in cervical adenocarcinoma are difficult to detect.^[9,10]^ It is likely that these are overlooked during the usual screening tests for cervical cancer. When cancer detection is delayed, the cancer generally is advanced when finally diagnosed and the prognosis may be unfavorable. We compared the SUVmax of FDG-PET/CT for primary staging in patients with cervical cancer and evaluated the relationship of the imaging findings to prognosis.

## 2 Materials and methods

### 2.1 Patients

We conducted a retrospective study of 34 patients with cervical cancer and 4 with carcinoma in situ (CIS)/adenocarcinoma in situ who underwent PET/CT examinations between April 2008 and March 2016 at our institution. Of the patients, 27 and 11 had squamous and glandular cancer, respectively. We excluded patients with conditions such as uncontrolled diabetes mellitus-related high blood sugar that could affect SUVmax value.^[11]^

PET/CT was not performed during the menstrual period to avoid physiologic FDG uptake. Patients were grouped according to disease stage, and the mean SUVmax, overall survival (OS), and progression-free survival (PFS) were evaluated.

This study was approved by the institutional review board of Kanazawa Medical University. All patients provided informed consent.

### 2.2 Classification of gynecologic cancer

Cervical cancer was classified according to the 2008 International Federation of Gynecology and Obstetrics (Fédération Internationale de Gynécologie et d’Obstétrique [FIGO]) staging system for cervical cancer.^[12]^ The results of CT, MRI, or PET examinations and the surgical-pathologic findings could not be used for staging classification although they were essential for treatment planning and might provide prognostic information.^[13]^

The samples were reviewed as part of routine clinical care by multiple pathologists. For all patients, treatment was performed according to Japan Society of Gynecologic Oncology guidelines 2011 for the treatment of uterine cervical cancer.^[14]^

### 2.3 Image acquisition conditions on PET/CT

The Biograph Sensation 16 scanner (Siemens, Bayern, Germany) was used to perform PET/CT. After at least 6 hours of fasting, patients were administered 185 MBq of FDG at an equivalent uniform dose intravenously. At 60 minutes after administration, a low-dose noncontrast CT scan for absorption correction was performed to collect anatomic information. The PET images had a matrix size of 256 × 256, which corresponded to a pixel size of 2.6 × 2.6 mm^2^. The PET data were reconstructed with an image resolution of approximately 6.5-mm full width at half maximum (FWHM). The PET images were reconstructed with an ordered-subset expectation maximization iterative reconstruction algorithm (8 subsets and 2 iterations).

**Figure 1:**
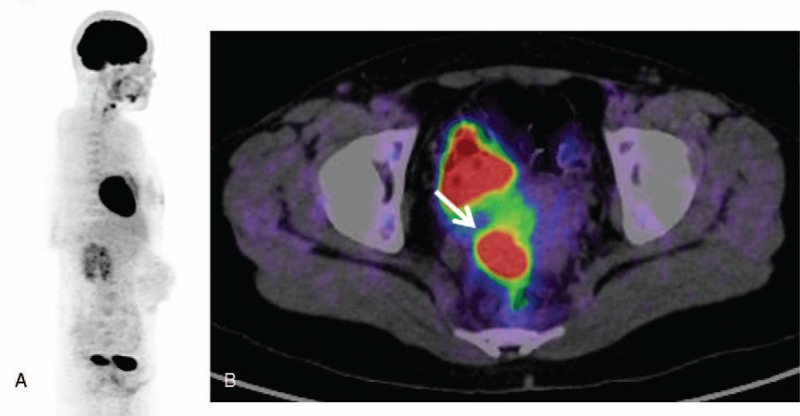
Positron emission tomography (PET)/computed tomography images of a patient with cervical cancer. (A) A typical whole-body 18F-fluorodeoxy-glucose (FDG)-PET image of a patient with cervical cancer. (B) A 32-year-old woman with stage IIA cervical cancer. FDG-PET images clearly show increased focal FDG accumulation (standardized uptake value = 22.43) in the tumor (arrow).

A PET/CT image of a typical case of cervical cancer is shown in Fig. 1.

### 2.4 Method for measuring SUV

SUVs were measured from regions of interest of the primary tumor. The SUV of a tissue sample was calculated as SUVmax in tissues in which FDG accumulation was confirmed. SUV was expressed using the following formula: radiation dose of the tissue (Bq/g)/[dose (Bq)/weight (kg)].

### 2.5 Statistical analysis

We measured and evaluated the difference in average SUVs between squamous and glandular cervical lesions. We used R version 3.2.4 (R Core Team, 2016) for statistical analyses. The level of statistical significance was set at *P* ≤ .05. The Mann–Whitney *U* test and receiver operating characteristic (ROC) curves were used to assess differences between 2 independent groups. Furthermore, ROC curve analysis included calculation of the area under the curve (AUC). The optimal cutoff value for sensitivity and specificity was determined using ROC curve analysis. The 5-year OS and PFS were calculated using the Kaplan–Meier method, and statistical significance was assessed using the log-rank test (*P* ≤ .05).

## 3 Results

### 3.1 Epithelial cervical tumors

A total of 27 patients (mean age, 54.5 ± 11.6 years; range, 32–74 years; SUV, 11.07 ± 17.72; range, 2.96–26.39) had squamous lesions and 11 (mean age, 55.0 ± 17.6 years; range, 34–87 years; SUV, 9.67 ± 7.78; range, 2.68–29.44) had glandular lesions. Mean SUVmax was not significantly different between these 2 groups (11.07 vs 9.67, respectively; *P* = .573).

### 3.2 Patients with squamous cervical cancer

Among the 27 patients with squamous lesions, mean SUVmax was significantly different between tumors <4 and ≥4 cm (5.87 vs 17.56, respectively; *P* < .001).

**Figure 2:**
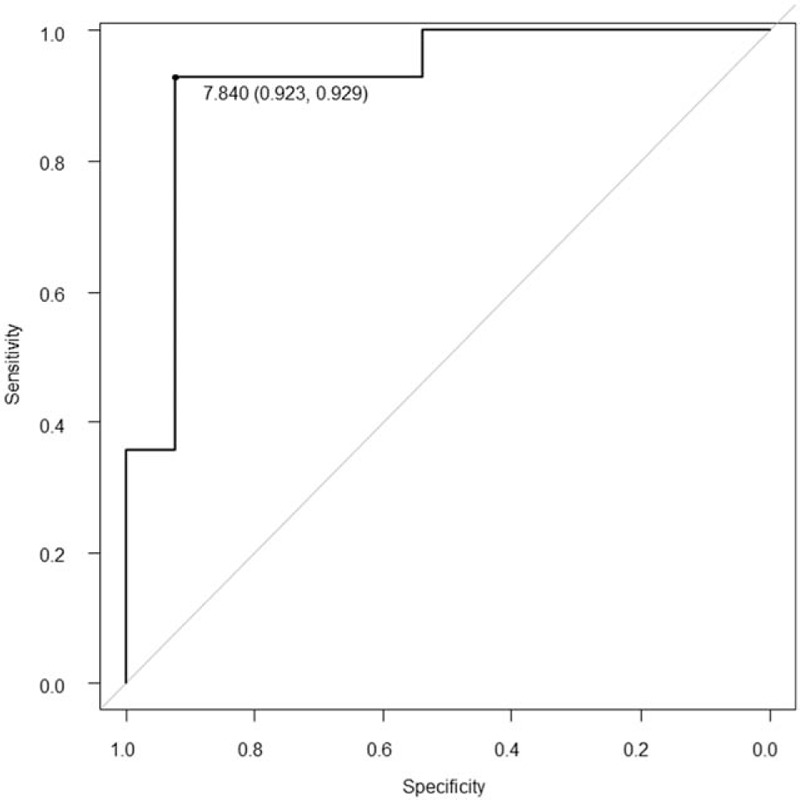
Receiver operating characteristic (ROC) curve comparison between stages ≤I and ≥II in 27 patients with squamous cervical cancer lesions. The ROC curve was the area under the curve = 0.923 with a 95% confidence interval of 0.811 to 1.000. A cutoff standardized uptake value of 7.84 was used.

We evaluated the staging system for cervical squamous lesions in 20 patients. Mean SUVs of 10 patients with stages I and 10 with stage II disease were 6.31 ± 4.62 and 14.35 ± 7.38, respectively (*P* = .003). There were no statistically significant differences in mean SUVs between other disease stages. Mean SUV of 13 patients with stage ≤I disease was significantly lower than that of 14 patients with stage ≥II disease (5.87 ± 4.17 vs 15.89 ± 7.14, respectively; *P* < .001). The ROC curve was AUC = 0.923 with a 95% confidence interval (CI) of 0.811–1.000. With a cutoff SUV of 7.84, the sensitivity and specificity were 92.9% and 92.3%, respectively (Fig. 2).

Among the 27 patients with squamous lesions, mean SUVmax was a tendency to significantly different between 21 patients without recurrence and 6 patients with recurrence (9.77 vs 15.63, respectively; *P* = .054).

**Figure 3:**
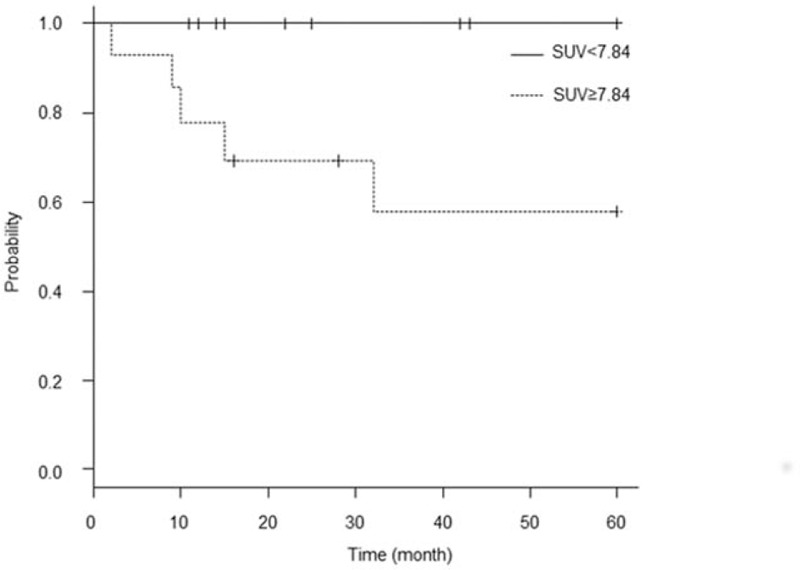
Kaplan–Meier curve for progression-free survival in 27 patients with squamous cervical cancer lesions. A log-rank test revealed a significant difference between the 2 curves (*P* = .020).

Among the 27 patients with squamous lesions and SUVs of <7.84 and ≥7.84 for OS and PFS, respectively, the log-rank test revealed a trend toward a significant difference between the 2 disease stages (*P* = .073 and *P* = .020, respectively; Fig. 3). The characteristics of squamous cervical tumors are shown in Table 1.

**Table 1:**
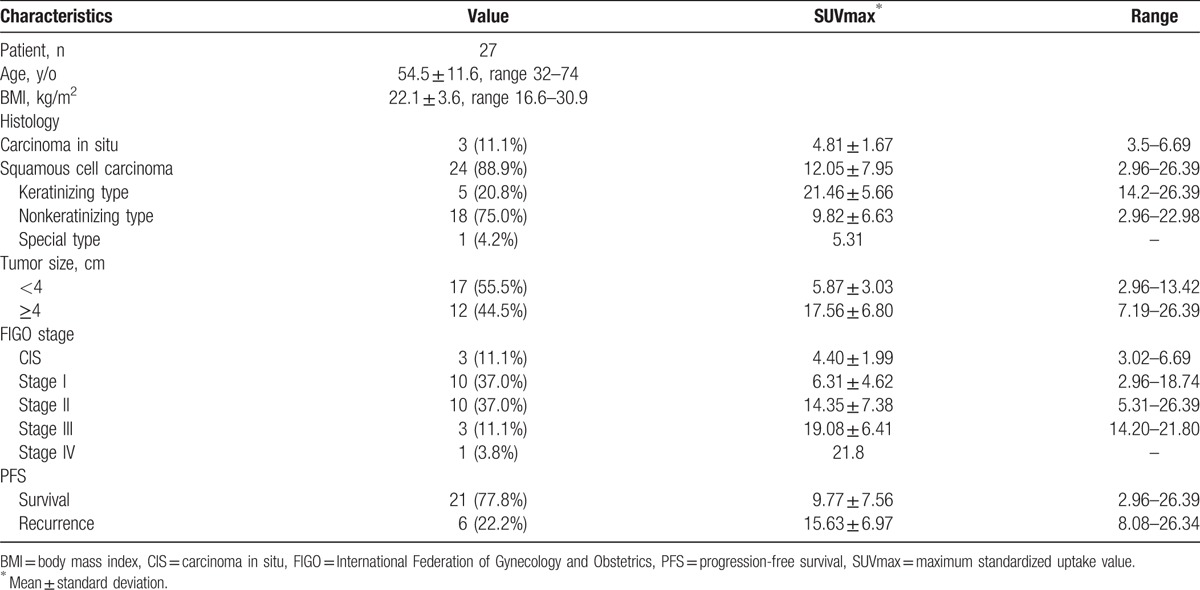
Characteristics of squamous cervical tumors.

### 3.3 Patients with glandular cervical cancer

**Figure 4:**
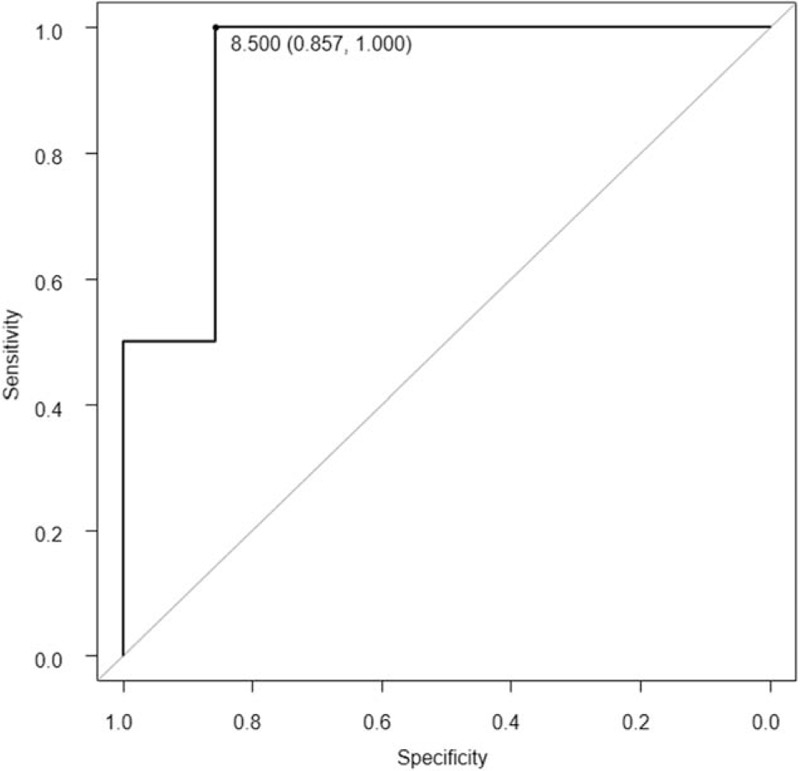
Receiver operating characteristic (ROC) curve comparison between stages ≤I and ≥II in 11 patients with glandular cervical cancer lesions. The ROC curve was the area under the curve = 0.929 with a 95% confidence interval of 0.767 to 1.000. A cutoff standardized uptake value of 8.50 was used.

Among the 11 patients with glandular lesions, the mean SUVmax was significantly different between tumors <4 and ≥4 cm (6.25 vs 18.80, respectively; *P* = .024). Comparison of the mean SUVs between each disease stage revealed no statistically significant differences. Mean SUVs of 7 patients with stage ≤I and 4 with stage ≥II disease were 5.92 ± 3.36 and 16.23 ± 9.44, respectively (*P* = .023). The ROC curve was AUC = 0.929 with a 95% CI of 0.767 to 1.000. With a cutoff SUV of 8.50, the sensitivity and specificity were 100% and 85.7%, respectively (Fig. 4).

Among the 11 patients with glandular lesions, mean SUVmax was significantly different between 7 patients without recurrence and 4 patients with recurrence (5.92 vs 16.23, respectively; *P* = .023).

**Figure 5:**
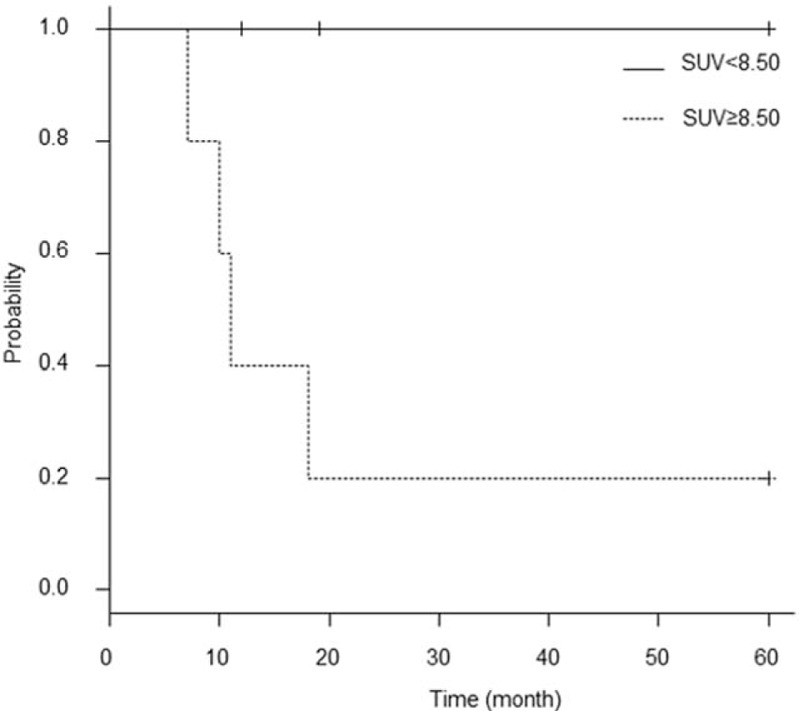
Kaplan–Meier curve for progression-free survival in 11 patients with glandular cervical cancer lesions. A log-rank test showed a significant difference between the 2 curves (*P* = .016).

With SUVs of <8.50 and ≥8.50 for OS and PFS, respectively, a log-rank test demonstrated no significant difference (*P* = 1.000) and a significant difference (*P* = .016) between the 2 disease stages, respectively (Fig. 5). The characteristics of glandular cervical tumors are shown in Table 2.

**Table 2:**
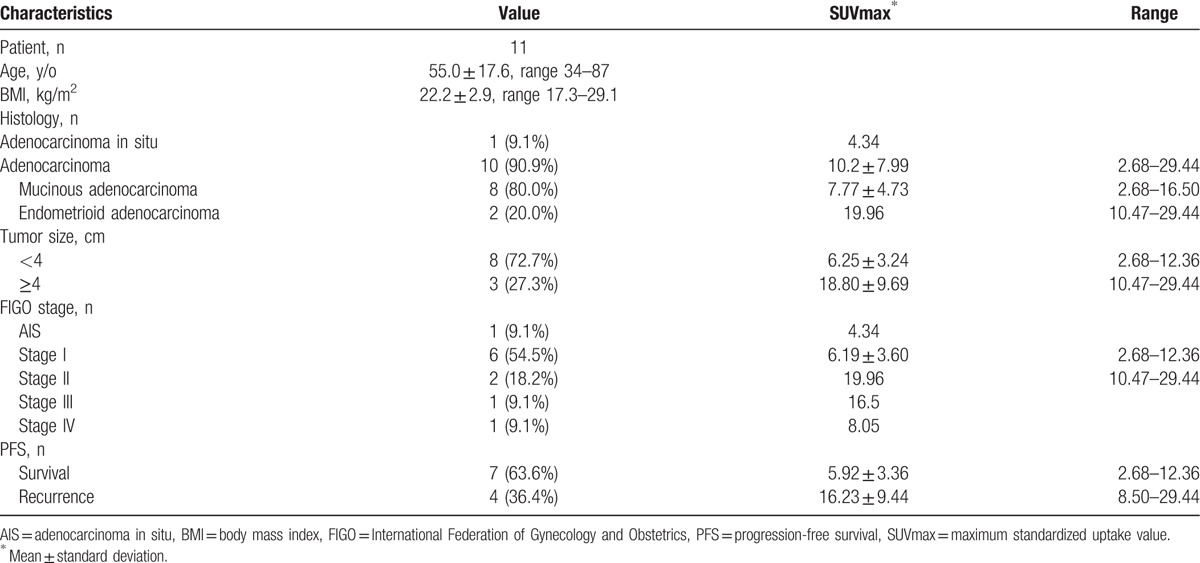
Characteristics of glandular cervical tumors.

## 4 Discussion

Our retrospective study aimed to investigate the efficacy of PET/CT for the diagnosis of early cervical cancer. We used the SUVmax of 38 patients with cervical lesions who underwent PET/CT to analyze whether there was a relationship among mean SUVmax, cervical cancer stage, and PFS.

Kidd et al^[15]^ reported a significant difference in the SUVmax for squamous versus nonsquamous tumors (*P* = .015). In a study of patients with nonsmall cell lung cancer, the mean SUVs of those with squamous cell carcinoma were higher than those with adenocarcinoma.^[16]^ Aquino et al^[17]^ reported significant differences in SUVmax between patients with adenocarcinoma and squamous cell carcinoma (*P* < .0001). At our institution, the mean SUVmax of patients with squamous cell carcinoma was higher than that of patients with adenocarcinoma, but the difference was not statistically significant. Patients with squamous carcinoma had many CIS and stage IA samples compared with those with adenocarcinoma. Accordingly, SUVmax might not have shown a significant difference due to sample bias. This may indicate that patients with squamous cell carcinoma tend to have a higher SUVmax than those with adenocarcinoma.

Lee et al^[18]^ reported that patients with early cervical cancer showing a high SUVmax (≥13.4) of the cervical tumor should be considered at increased risk for disease recurrence postoperatively. Wagner et al^[19]^ reported that the new FIGO staging system for cervical cancer, with the inclusion of size >4 cm for stage IIA cancers, better reflects survival and overall prognosis. Also, Kyung et al^[20]^ reported that tumor size (≤4 vs 4–6 cm, *P* = .0371; and ≤4 vs >6 cm, *P* = .0024) was identified as an independent predictive factor for the prognosis of stage II to IV cervical cancer. At our institution, the mean SUVmax was significantly different between tumors >4 and ≤4 cm in squamous and glandular cervical cancers. The SUVmax may become the important factor as a prognosis evaluation of cervical cancer.

Chou et al^[21]^ reported low sensitivity of FDG-PET for patients with stage IA2 to IIA cervical cancer. Yu et al^[22]^ reported that the uptake of early-stage cervical carcinoma showed no statistical significance between groups with stage IB and IIA diseases (*P* > .05). In contrast, Chung et al^[23]^ reported that median preoperative SUVmax values in the primary tumors were significantly higher in patients with higher FIGO stages (*P* = .0149). In our study, the mean SUVmax in patients with stage ≤I and ≥II diseases demonstrated a strongly significant difference, suggesting that use of the mean SUVmax is feasible for differentiating between stage ≤I and ≥II cervical cancer.

Regarding the recurrence of cervical cancer, the cure rate after conization of CIS is reported to be approximately 100%,^[24–26]^ Approximately 95% of patients with stage IA cervical cancer survive without any evidence of cancer recurrence 5 years after surgery or radiation therapy.^[27]^ Perez et al^[28]^ reported recurrence rates of approximately 10%, 17%, 23%, 42%, and 74% for stage IB, IIA, IIB, III, and IVA diseases after radiotherapy alone, respectively. The recurrence rate of cervical cancer ranges between 11% and 22% for FIGO stages IB to IIA and between 28% and 64% for FIGO stages IIB to IVA.^[29]^ Regarding the survival rate of cervical cancer, Yagi et al^[30]^ reported that SUVmax of the primary tumor on preoperative FDG-PET/CT is a prognostic indicator in patients with stage IA2 to IIB cervical cancer treated with radical hysterectomy. At our institution, we divided our study patients with squamous cell carcinoma and adenocarcinoma into separate groups using an SUVmax cutoff point between stage ≤I and ≥II diseases and demonstrated a significant difference between both in terms of PFS using the log-rank test. This significant difference in SUVmax between disease stages suggested that this measurement can be used to predict disease recurrence.

Our study had several limitations. Surgical treatment strategies were different for stage IA1 and IA2 cervical cancer. Conization or total hysterectomy was recommended for the former, whereas extended hysterectomy or radical hysterectomy was recommended for the latter. However, we had no patient with stage IA cancer; therefore, the significance of SUV for this stage was not understood in our study. SUV values differed according to the administration of uniform 185-MBq FDG and blood glucose levels of the subject. Furthermore, PET/CT may show a bias in detecting early cervical cancer lesions at approximately <6.5-mm FWHM. The clinical significance including these very advanced cases must be questioned.

## 5 Conclusion

SUVmax from FDG-PET/CT is feasible for differentiating between clinical stage ≤I and ≥II cervical cancer. Moreover, SUVmax is suggested to be useful for the prognostic evaluation of disease recurrence in patients with cervical cancer.

## Acknowledgment

The authors would like to thank Enago (www.enago.jp) for the English language review.
